# Supplementary material for: Iterative improvement in the automatic modular design of robot swarms
Source: PeerJ Comput Sci. 2020 Dec 7;6:e322. doi: 10.7717/peerj-cs.322 (PMC7924708; doi:10.7717/peerj-cs.322)
Supplement: Supplemental Information 3 [file peerj-cs-06-322-s003.zip › argos3/doc/api/standalone/a00459_source.html]

ARGoS: plugins/robots/foot-bot/simulator/footbot\_distance\_scanner\_default\_actuator.h Source File


- Main Page
- Related Pages
- Namespaces
- Classes
- Files

- File List
- File Members

# plugins/robots/foot-bot/simulator/footbot\_distance\_scanner\_default\_actuator.h

Go to the documentation of this file.

```
00001 
00007 #ifndef FOOTBOT_DISTANCE_SCANNER_DEFAULT_ACTUATOR_H
00008 #define FOOTBOT_DISTANCE_SCANNER_DEFAULT_ACTUATOR_H
00009 
00010 #include <string>
00011 #include <map>
00012 
00013 namespace argos {
00014    class CFootBotDistanceScannerDefaultActuator;
00015 }
00016 
00017 #include <argos3/plugins/robots/foot-bot/control_interface/ci_footbot_distance_scanner_actuator.h>
00018 #include <argos3/plugins/robots/foot-bot/simulator/footbot_entity.h>
00019 #include <argos3/plugins/robots/foot-bot/simulator/footbot_distance_scanner_equipped_entity.h>
00020 #include <argos3/core/simulator/actuator.h>
00021 
00022 namespace argos {
00023 
00024    class CFootBotDistanceScannerDefaultActuator : public CSimulatedActuator,
00025                                                   public CCI_FootBotDistanceScannerActuator {
00026 
00027    public:
00028 
00029           static const Real RPM_TO_RADIANS_PER_SEC;
00030 
00031       CFootBotDistanceScannerDefaultActuator();
00032       virtual ~CFootBotDistanceScannerDefaultActuator() {}
00033 
00034       virtual void SetRobot(CComposableEntity& c_entity);
00035 
00036       virtual void SetAngle(const CRadians& c_angle);
00037       virtual void SetRPM(Real f_rpm);
00038 
00039       virtual void Enable();
00040       virtual void Disable();
00041 
00042       virtual void Update();
00043       virtual void Reset();
00044 
00045    private:
00046 
00047       CFootBotDistanceScannerEquippedEntity* m_pcDistanceScannerEquippedEntity;
00048 
00049       CRadians m_cDesiredRotation;
00050       Real     m_fDesiredRotationSpeed;
00051       UInt8    m_unDesiredMode;
00052 
00053    };
00054 
00055 }
00056 
00057 #endif
```

---

Generated on 10 Jul 2018 for ARGoS by 
 1.6.1
